# Testing the adaptive value of gastropod shell morphology to flow: a multidisciplinary approach based on morphometrics, computational fluid dynamics and a flow tank experiment

**DOI:** 10.1186/s40851-018-0119-6

**Published:** 2019-01-18

**Authors:** Gerlien Verhaegen, Hendrik Herzog, Katrin Korsch, Gerald Kerth, Martin Brede, Martin Haase

**Affiliations:** 1 Vogelwarte, Zoologisches Institut und Museum, Universität Greifswald, Soldmannstraße 23, 17489 Greifswald, Germany; 2 Institut für Zoologie, Rheinische Friedrich-Wilhelms-Universität Bonn, Meckenheimer Allee 169, 53115 Bonn, Germany; 3 Angewandte Zoologie und Naturschutz, Zoologisches Institut und Museum, Universität Greifswald, Loitzer Str. 26, 17489 Greifswald, Germany; 4 Lehrstuhl Strömungsmechanik, Universität Rostock, Albert-Einstein-Str. 2, 18051 Rostock, Germany

**Keywords:** *Potamopyrgus antipodarum*, Computational fluid dynamics, Flow tank, Morphology, Adaptation, Gastropod

## Abstract

A major question in stream ecology is how invertebrates cope with flow. In aquatic gastropods, typically, larger and more globular shells with larger apertures are found in lotic (flowing water) versus lentic (stagnant water) habitats. This has been hypothetically linked to a larger foot, and thus attachment area, which has been suggested to be an adaptation against risk of dislodgement by current. Empirical evidence for this is scarce. Furthermore, these previous studies did not discuss the unavoidable increase in drag forces experienced by the snails as a consequence of the increased cross sectional area. Here, using *Potamopyrgus antipodarum* as a study model, we integrated computational fluid dynamics simulations and a flow tank experiment with living snails to test whether 1) globular shell morphs are an adaptation against dislodgement through lift rather than drag forces, and 2) dislocation velocity is positively linked to foot size, and that the latter can be predicted by shell morphology. The drag forces experienced by the shells were always stronger compared to the lift and lateral forces. Drag and lift forces increased with shell height but not with globularity. Rotating the shells out of the flow direction increased the drag forces, but decreased lift. Our hypothesis that the controversial presence of globular shells in lotic environments could be explained by an adaptation against lift rather than drag forces was rejected. The foot size was only predicted by the size of the shell, not by shell shape or aperture size, showing that the assumed aperture/foot area correlation should be used with caution and cannot be generalized for all aquatic gastropod species. Finally, shell morphology and foot size were not related to the dislodgement speed in our flow tank experiment. We conclude that other traits must play a major role in decreasing dislodgement risk in stream gastropods, e.g., specific behaviours or pedal mucus stickiness. Although we did not find globular shells to be adaptations for reducing dislodgement risk, we cannot rule out that they are still flow-related adaptations. For instance, globular shells are more crush-resistant and therefore perhaps adaptive in terms of diminishing damage caused by tumbling after dislodgement or against lotic crush-type predators.

**Electronic supplementary material:**

The online version of this article (10.1186/s40851-018-0119-6) contains supplementary material, which is available to authorized users.

## Background

A major question in stream ecology is how invertebrates cope with flow [1]. It is indeed long known that many stream species avoid exposing themselves to flow [2] or exhibit morphological adaptations when compared to lentic (i.e. stagnant waters) species (e.g. [1, 3]). The main selective pressures involved in adaptation to flow were later identified to be 1) drag and 2) lift forces, 3) corrasion, and 4) diffusion through boundary layers [4]. Drag (F_D_) (**Eq.** **1**) is the force acting on an object due to the impingement of the fluid, while lift (F_L_) (**Eq.** **2**) is the vertical force acting perpendicularly to the relative flow direction resulting in a difference of pressures on opposites sides of the object. Both forces are defined as follows:

1 $$ {F}_D={C}_D\rho A\frac{v^2}{2} $$

2 $$ {F}_L={C}_L\rho A\frac{v^2}{2} $$

with ***C***_***D***_ being the drag coefficient, ***ρ*** the fluid density, ***A*** the projected frontal surface area of the object, ***V*** the velocity (object speed relative to flow speed), and ***C***_***L***_ the lift coefficient. ***C***_***D***_ is composed of the effects of skin friction drag, i.e. the drag depending on the smoothness of the surface of an object, and form drag, i.e. the drag depending on the shape of the object. High drag and lift can impede the active movement of stream living organisms and increase dislodgement risk. While it is difficult to assess the biological significance of the energy costs for organisms to counter drag (e.g. [5]), it is undisputed that dislodgement can lead to physical damage and may redeposit organisms to less suitable habitats (e.g., [6]), although little is known about the fate of organisms once carried away. The third selective pressure, corrasion, is defined as the risk of abrasion through suspended solids [7]. In contrast to these risk-increasing factors, the diffusive exchange processes of e.g. gas or ions through the thinner boundary layers are usually enhanced in lotic (i.e. flowing water) versus lentic habitats and thus are a positive factor of living in streams.

These four selective pressures all depend on the interplay between the fluid properties and the shape and size of the organisms. As a result, disentangling their relative significance in the morphological adaptation of organisms to flow is a complex matter. For instance, Weissenberger et al. [8] showed striking differences in the relative importance of drag and lift forces across different species of mayflies and stoneflies studying living larvae in a flow tank at equal velocities. Furthermore, morphologies favouring flow separation, i.e. the detachment of flow from the body surface of the organisms, would on the one hand decrease corrasion risk, but on the other hand would also negatively influence the diffusive exchange processes as the water renewal near the body surface would decrease as well [4]. Thus, organisms cannot simultaneously optimize their morphology with respect to all four selective pressures. The final morphology will necessarily be the result of evolutionary trade-offs.

In the last decades, stream ecology has benefitted from technological innovations and improved measurement methods, although this has also revealed the complexity of hydrodynamic adaptation, making generalizations for the diversity of invertebrates difficult [1]. Shells of aquatic gastropods are ideal objects to study adaptation to flow as these hard structures are permanently exposed to their liquid environment. Typically, larger and wider but shorter (globular) shells with larger apertures are found in lotic versus lentic habitats (e.g. in *Bellamya* spp. [9], *Radix labiata* (as *Lymnaea peregra* [10]), *Lithasia* spp. [11, 12], and *Elimia potosiensis* [13]).

It is commonly suggested that a larger aperture in gastropods, associated with a larger foot and thus attachment area, is an adaptation against risk of dislodgement by current (e.g. [14]). Empirical evidence for this is scarce, though. Increased adhesion due to larger aperture and foot size was shown e.g. for the marine gastropod *Nucella lapillus* in habitats with higher wave exposure [15]. However, these studies did not discuss the unavoidable increase in drag forces experienced by the snails as a consequence of the increased cross-sectional area, thus some of these morphological structures believed to be adaptations to resist water flow may increase dislodgement risk, not reduce it. The first attempts to link shell morphology of freshwater snails to dislodgement speed and experienced drag forces were done by the use of flow tanks and Newton meters (e.g. [16–18]). Although drag forces increased with shell size, which had a higher impact on dislodgement speed than foot size, the relationship between shell size and dislodgement speed varied highly among species. Laser Doppler Anemometry was later used to investigate velocity gradients around gastropod shells aiming at a better understanding of the relationships between the morphology and drag, lift, corrasion and diffusive exchanges [4].

Recently, the New Zealand mud snail *Potamopyrgus antipodarum* (Grey, 1843) has emerged as a promising model to study hydrodynamic adaptation as it occurs in a wide range of habitats, including all types of freshwater environments and brackish estuaries, thereby exhibiting an extreme variability in shell shape, size and armature [19–23]. Moreover, *P. antipodarum* is ovoviviparous and has successfully invaded four continents during the last 200 years [24–28]. Unlike in *P. antipodarum*’s native range, where obligate diploid sexual and obligate polyploid asexual individuals coexist [29, 30], only asexually reproducing individuals are found in the invasive range [31–34]. A number of recent studies have revealed complex interactions between habitat characteristics including flow, shell shape, size, and fitness in this species [22, 23, 35–37]. It appears that flow imposes a number of counteracting selective forces on shell characteristics resulting in evolutionary trade-offs. For example, lower frequencies of spiny snails were found in streams as compared to lakes, presumably because spiny shells tend to collect seston, i.e., matter floating in the water body, increasing the drag of the shell, although spines may protect against predation [38]. In this species as well, the typically larger but more globular shell morphology with larger aperture area was found at higher flow rates, the increase in drag being hypothesized to be counterbalanced by a larger foot [22, 23, 36, 37]. However, at higher flow rates, shells with larger aperture areas relative to the shell size as well as snails with relatively slender shells had higher brood sizes, thus seemed better adapted in terms of fitness compared to snails with globular shells. In general, larger snails always showed higher fecundity, although the relationship between size and fecundity was weaker with increasing flow rate in invasive as compared to native populations, reflecting again possible trade-offs in exhibiting a larger and globular shell in lotic habitats [22, 23].

In the present study, we sought to better understand the hydrodynamic adaptation of shell morphology in gastropods, using *P. antipodarum* as study model by integrating computational fluid dynamics (CFD) simulations and a flow tank experiment with living snails. Although evolutionary trends are easier to detect at higher taxonomic levels, studying adaptation on the intraspecific level does not need to consider phylogenetic constraints [39] and is therefore especially well-suited to identify adaptive pressures and provide insights to the microevolutionary mechanisms leading to phenotypic differentiation [40, 41]. Here, we used the CFD simulations to calculate the relative drag and lift forces of three shell morphologies (globular, intermediate, and slender), and tested the overall hypothesis that shell morphology in gastropods is an adaptation against dislodgement through lift rather than drag forces, the latter inevitably increasing with diameter. This would explain the counterintuitive presence of wider shells with shorter spires in lotic environments. We complemented our CFD simulations, which could only test forces on the shells without the presence of the soft body, with a flow tank experiment. Here, we tested the specific hypothesis that the dislocation velocity of living snails is linked to shell morphology and foot size, and that the latter can be predicted by shell morphology, in particular the aperture area as assumed by several authors [22, 23, 36, 37].

## Methods

In this study, we combined analyses of behaviour with CFD simulations. We used μ-CT-scanned mollusc shells to obtain 3D models that were used for CFD simulations to calculate the hydrodynamic forces. The results of these theoretical models were then compared with our behavioural study conducted in a flow tank. In both, naturally experienced flow velocities were applied [22, 23].

### Shell model generation

We based our models on three *P. antipodarum* snails chosen for their distinct shell shapes: snail 1 was short and wide, snail 3 narrow and elongate, and snail 2 had an intermediary shell shape (Table 1). Snail 1 was collected from an approximately 8 m-wide river in the Kaniwhaniwha reserve in the Waikato region on New Zealand’s North Island (S 37° 55′ 12.8", E 175° 4’ 52.9") in February 2016; snail 2 from Lake Kiessee, near Jarmen in Mecklenburg-Western Pomerania in NE Germany (N 53° 55′ 44.5", E 13° 18’ 60.0") in June 2017; and snail 3 from an approx. 1 m-wide river in the northern West Coast region of New Zealand’s South island (S 42° 2′ 9.3", E 171° 23’ 21.1") in March 2016. All snails were fixed on site in 70% ethanol. Snails retract upon contact with ethanol; therefore, our models were based on the shells only.

**Table 1:** Shell measurements of the original snails used in the computational fluid dynamics simulations

	Shell height (mm)	Shell width (mm)	Aperture height (mm)	Aperture width (mm)
Snail 1	4.92	3.10	2.13	1.89
Snail 2	4.37	2.44	1.59	1.49
Snail 3	4.01	1.98	1.35	1.21

**Fig. 1:**
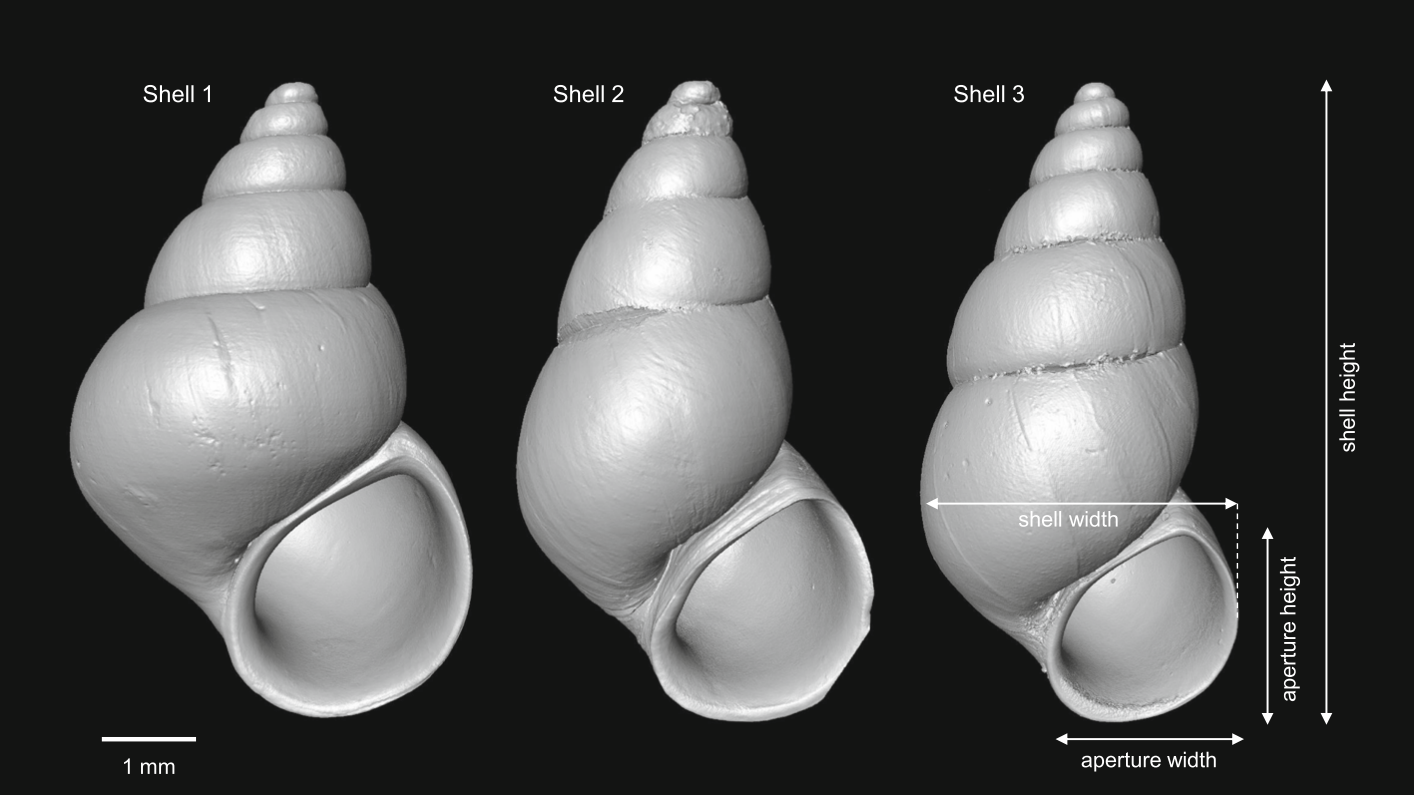
Three-dimensional surface models obtained from μCT. Shells 2 and 3 were scaled to the same height as shell 1

The three shells were mounted vertically by gluing the apex onto a pin and scanned with a μCT Xradia MicroXCT-200 scanner (Carl Zeiss X-ray Microscopy Inc., Pleasanton, USA) at 40 kV, 8 W and at four times magnification. The pixel sizes for the three snail shells were 5.087, 3.981 and 4.031 μm, respectively. The image stacks (in TIFF format) were processed and 3D surfaces models constructed with an isosurface threshold of ~ 35,000 with the AMIRA v. 5.6.0 (FEI, Visualization Science Group) software (Fig. 1). The final models were exported as a stereolithography (*.stl) file and further processed in the Meshlab software (64-Bit Version 1.3.3 Visual Computing Lab – ISTI – CNR, http://meshlab.sourceforge.net/). In Meshlab, redundant details of the shell, i.e. the inner structures, were removed from the model by ambient occlusion filtering and a new mesh surface was created on the remaining vertices by means of Poisson surface reconstruction. The models were scaled according to real world dimensions by voxel size given by the μCT reconstruction software. These completely closed polygon models contained about 11,000–16,000 faces (model 1: 12,686; model 2: 16,280; model 3: 11,774), were exported as polygon meshes (*.stl), and then used for the CFD simulation.

### CFD setup

**Fig. 2:**
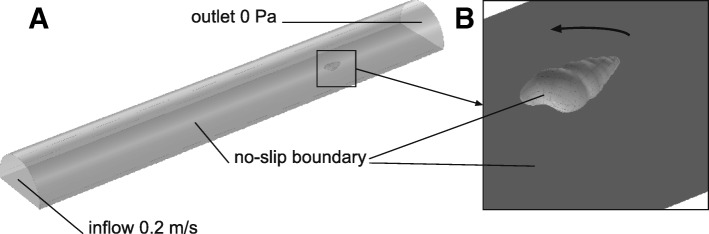
Experimental setup used for the CFD studies. **a** semi-circular flow channel resembling the channel with the platform where the snails were placed in the real experiments. Flow was applied from left to right. Beside the inflow and outlet facets of the tank, all boundaries were set to a no-slip condition, i.e. a boundary layer was formed on these faces. **b** close-up of the shell placed just above the platform. The shell was rotated anticlockwise in the experiments as indicated by the circular arrow

**Fig. 3:**
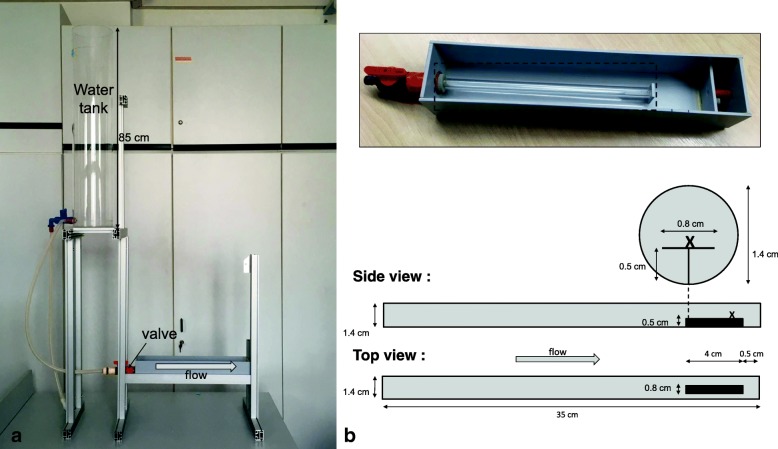
Flow tank setup (**a**) and close-up of the cylindrical pipe (**b**). Cross = initial position of snails

CFD was performed using the commercial software ANSYS CFX v. 18.0 (ANSYS Inc.) which solves the time averaged equations of motion on unstructured grids. The virtual experiments were conducted in a half-cylindrical water column (Fig. 2) built in ANSYS ICEM CFD v. 18.0 mimicking the real experimental channel (Fig. 3): diameter 14 mm, length 100 mm with a platform at the bottom section. Five shell models were tested in total: shells 1–3 in their original dimensions, and then again shell 2 and 3 scaled to the height of shell 1 (Fig. 1) to compensate for the differences in size between the shells (Table 1). The shell models were loaded into the simulation environment and placed in comparable position and orientation into the virtual flow channel (Fig. 2). To investigate the influence of the orientation of the snail to the flow, the angle between the main axis of the shell and the main flow direction was altered horizontally anticlockwise (0°, 45° and 90°). For every shell (shell 1, shell 2, shell 3, scaled shell 2 and scaled shell 3) at every orientation, a tetrahedral mesh of the set up was created according to the octree meshing algorithm. After removing the volume mesh of the shell models, the “hollow” meshes were exported as binary (*.cfx5) format. The final meshes are containing approximately 750,000 number of elements.

The created meshes were used for the CFD simulations in CFX with a root mean square (RMS) residual target of the equation system of 0.0001 and an incoming water flow velocity of 0.2 m/s (Re = 2793.6). To study how the flow environment around the shells and the lift-on-drag ratio would vary with increasing flow, simulations for the shell models 1–3 at 0° rotation were repeated at 0.6 m/s (Re = 8380.7) and 1 m/s (Re = 13,967.9), to cover the velocity range of the flow tank experiment. The Reynolds numbers (Re) of the flow in the channel and for the shell models in our experimental set-up were calculated based on **Eq.** **3**:

3 $$ \operatorname{Re}=\frac{uD}{v} $$

with **D** being the diameter of the cylinder for the flow in the channel, or the hydraulic diameter (**D**_**H**_) in the case of the shell models, ***u*** the mean fluid velocity, and ***ν*** the kinematic viscosity of the fluid (here, ***ν*** = 10^− 6^ for water at 20 °C). The Reynolds number is a dimensionless quantity used to predict the transition from laminar to turbulent flow, e.g. for a bluff body. The boundary layer, which governs the experienced drag and lift forces by its length and thickness, stays laminar up to Re = 200,000, whereas for a slender body, the transition Re number is even higher [42]. As the shape of a shell lies between the shape of a bluff and slender body, the Reynolds numbers, for all our simulations, which varied between 2793 and 13,967 in the channel and between 396 and 3100 for the shell models (Additional file 1: Table S1), were well below the transition range. Thus, the turbulence option for the incoming flow was set as laminar in all the simulations, and the flow models were based on the Navier-Stokes equation [43], without the need to introduce a turbulence model. For more general information on CFD techniques see [44–47].

Drag, lift and lateral forces experienced by the shell models for every CFD simulation were obtained by the integration of the pressure and viscous forces in x, y and z direction. The cross-sectional areas (A) of the shells were obtained in ANSYS for every set up and used to calculate the dimensionless drag (C_d_) and lift coefficients (C_L_) by transforming Eqs. 1 and 2. The influence of the flow velocity on the drag, lift, lateral forces and the lift-on-drag ratio experienced by the shells at 0° rotation was tested with four distinct correlations and the flow fields around the shells were visualized in ANSYS CFX-Post. The influence of the shell orientation towards the flow on the drag, lift, lateral forces, and the cross-sectional area was then tested in four distinct correlations. Finally, the correlation of the lift-on-drag ratio with shell height, shell shape (height/width), and the rotation angle was tested.

### Flow tank experiment

The speed of the water at which snails were detached was determined for a total of 80 individuals from four populations: two lake populations, Dobersdorfer See (Schleswig Holstein, Germany, N 54° 19′ 51.8", E 10° 17’ 4.3", *N* = 20), and the Kiessee near Jarmen (Mecklenburg-Western Pomerania, Germany, N 53° 55′ 44.5", E 13° 18’ 60.0", N = 20); and two spring brook populations, Quellsumpf Ziegensteine (island of Rügen, Germany, N 54° 21′ 23.7", E 13° 36′ 27.0", *N* = 21), and a spring draining into Waitewheta River along Franklin Road (Waikato, New Zealand, S 37° 27′ 47.1", E 175° 46’ 48.1", *N* = 19). Specimens were either adult as indicated by a fully developed apertural lip [22] or subadult. All snails were kept in aquaria in artificial pond water (APW, 0.5 g/L sea salt, Tropic Marine®, Germany) in a climate cabinet at 18 °C and 16/8 h light/dark cycle. The snails from Dobersdorfer See were originally collected in summer 2015, the New Zealand population in March 2016, and the remaining two populations in May 2017, one month before the experiments. Hence, the snails from the first two populations used in the experiment were offspring of the collected ones. All four populations were clonal [22, 23].

The flow tank consisted of a 35 cm long acrylic tube with 1.4 cm inner diameter. The front end was connected to a water reservoir via a valve that was operated manually. We glued a strip of graph paper to it in order to define accurate opening positions. The rear end of the tube had a small, elevated platform of 4 cm lengths onto which the snails would be placed so that they would be exposed to the centre of the water jet. The entire tube was mounted in a box where it could always stay submerged and filled with water, even when there was no flow from the reservoir to allow the placement of snails. The whole apparatus is depicted in Fig. 3.

In order to measure the speed of the water jet, a calibration was first made by placing a ball furled of aluminium foil in water in order to prevent air inclusions and with a diameter of approx. 5 mm onto the anterior end of the platform. Before opening the valve, the conducting hose was kinked and the water thus blocked. This allowed releasing the water suddenly with hardly any delay at a given position of the valve. The ball was pushed along the platform and filmed with a Miro LC320 camera with 2000 frames per second. The speed was calculated from the number of frames the ball needed to pass the final, marked centimetre. This was done for eight different valve positions and repeated three times for each position. The maximum value at each position was taken to construct a calibration speed curve.

As the required water volume exceeded our storage capacity of APW, the experiments were conducted in tap water of 18 °C which apparently did not influence the behaviour of the snails of these four populations as tested beforehand in a pre-trial in contrast to other populations. A snail was placed onto the rear end of the platform. The valve was then opened at the first position to apply a weak current which induced the snail to orient against the flow direction. Once it had assumed this orientation and started to crawl against the flow the valve was steadily opened to increase the flow. The valve position at which the snail was detached was recorded. The snails were placed in random order. The valve was operated only by one of us, KK, after foregoing calibration trials controlling the opening speed with a digital clock. The variation of the opening time remained remarkably small throughout the experiment ranging from 13.01 to 13.40 s (3%) to open fully. The water level in the reservoir was kept constant by another operator.

After the snails had been detached and washed out of the tube, they were individually placed into small Petri dishes and back in the climate cabinet to await the measurement of the foot area on one of the following days. In order to do so, the snails crawling in their Petri dishes were photographed three times from underneath through a microscope objective of 1.3 x magnification fitted to a digital ToupTek DCM510 camera. The foot area was then measured using NIH ImageJ. For statistical analyses, the largest area measured for each foot was retained. Subsequently, the snails were fixed in 96% ethanol and the shells photographed under a Carl Zeiss Discovery V20 microscope equipped with an AxioCam MRc camera and a Plan Apo S 0.63x objective to measure shell height and width, aperture height and width, all in Carl Zeiss AxioVision v. 4.8, and the area of the aperture, again with ImageJ.

Student’s *t*-tests were used to compare shell height, shell shape (shell height/width), and the aperture and foot areas corrected for shell height between the two habitat types (lake or spring brook) of the origins of the snails. The perture area was correlated to shell height, shell width, and shell shape. Using a linear model (LM) we aimed at explaining the foot size by shell height and shape, the interaction of shell height and shape, aperture area, habitat type, population origin nested in habitat type and the interaction of aperture area and population origin as potential explanatory variables. To normalize the distribution of the foot size, the values were first inverted. In another LM the dependence of the dislodgement speed on shell height or shape, foot size, the interactions of foot size with shell height and shape, aperture surface, habitat or population origin was tested. Shell width was not included as variable in the LM analyses as it was positively correlated with shell height (Kendall’s *Tau* = 0.787, *P* < 0.001).

All statistical tests in this study were executed in PAST v.3.14 [48] or in R v.3.3.3 [49]. All LMs were run in R with the *lme4* v.1.1–13 package [50] and built by dropping terms based on Chi-square tests using the drop1 function [51]. Non-parametric tests were used if normal distributions were rejected by a Shapiro-Wilk test.

## Results

### CFD

Results from the CFD simulations at 0.2, 0.6 and 1 m/s flow velocity are found in (Table 2).

**Table 2:** Results from the computational fluid dynamics simulations

Shell model	Flow velocity (m/s)	Rotation angle (°)	Cross sectional area (mm^2^)	Drag (μN)	Lift (μN)	Lateral force (μN)	Lift/drag	Drag coefficient C_D_	Lift coefficient C_L_
1	0.2	0	5.461	104.2	82.2	10.6	0.789	0.956	0.754
2	0.2	0	2.780	45.2	23.3	5.7	0.514	0.814	0.419
2 scaled	0.2	0	3.525	59.8	34.1	8.4	0.570	0.850	0.485
3	0.2	0	2.459	37.6	19.9	4.0	0.530	0.765	0.406
3 scaled	0.2	0	3.701	64.5	35.8	7.3	0.555	0.873	0.485
1	0.2	45	5.713	98.6	69.7	−26.7	0.706	0.865	0.611
2	0.2	45	3.342	52.7	10.8	−17.4	0.205	0.790	0.162
2 scaled	0.2	45	4.236	71.2	15.0	−24.0	0.211	0.842	0.178
3	0.2	45	3.263	50.0	12.2	−19.6	0.244	0.768	0.188
3 scaled	0.2	45	4.911	84.3	20.6	−33.4	0.244	0.860	0.210
1	0.2	90	7.083	148.7	39.2	−36.1	0.264	1.051	0.277
2	0.2	90	4.481	88.5	4.4	−22.2	0.050	0.990	0.050
2 scaled	0.2	90	5.679	121.8	5.9	−31.6	0.048	1.074	0.052
3	0.2	90	4.321	83.1	4.2	−19.4	0.050	0.963	0.048
3 scaled	0.2	90	6.504	144.1	4.9	−33.5	0.034	1.110	0.038
1	0.6	0	5.461	714.4	651.3	119.8	0.912	0.728	0.664
2	0.6	0	2.780	296.1	198.1	40.1	0.669	0.593	0.396
3	0.6	0	2.459	270.6	193.0	19.4	0.713	0.612	0.437
1	1	0	5.461	1861.4	1772.9	370.8	0.952	0.683	0.650
2	1	0	2.780	744.3	611.7	193.1	0.822	0.536	0.441
3	1	0	2.459	709.0	621.1	180.3	0.876	0.578	0.506

### Drag and lift at 0° orientation

**Fig. 4:**
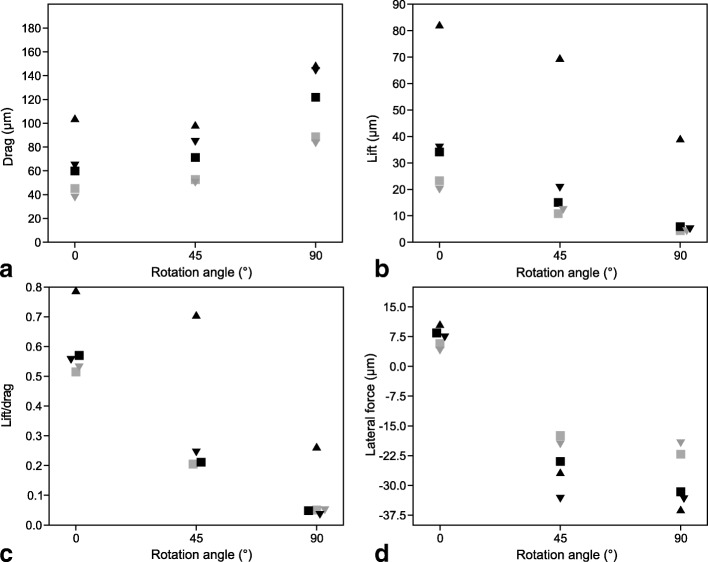
Drag forces (**a**), lift forces (**b**), lift-on-drag ratio(**c**) and lateral forces (**d**) exerted on the shell models at 0.2 m/s flow rate at the different rotation angles in the computational fluid dynamics simulations. Triangle = shell 1; square = shell 2; inversed triangle = shell 3; black = models scaled to same height as shell 1; grey = original size. Overlapping symbols were slightly displaced along the x axis to increase their visualization

In their original size and at flow velocity of 0.2 m/s as well as 0° orientation, drag and lift forces decreased, as expected, from shell 1 to shell 3 (Fig. 4a, b). When scaled to the height of shell 1, drag (104.2 μN) and lift (82.2 μN) forces on shell 1 were approximately twice as high as on shells 2 and 3, which experienced similar drag (59.8 and 64.5 μN, respectively) and lift forces (34.1 and 35.8 μN, respectively) (Fig. 4a, b). The lift coefficient C_L_ showed a similar pattern, with the highest value for shell 1 (0.754) and identical values for the scaled shells 2 and 3 (0.485). Although the highest drag coefficient C_D_ was also found for shell 1 (0.956), the difference to the scaled shell 2 (0.850) and shell 3 (0.873) was not so pronounced. Drag forces were on average 1.7 times higher than lift forces (mean drag-on-lift ratio = 1.731 ± 0.269) and 8.6 time higher than lateral forces (8.551 ± 1.278). Drag (Kendall’s *Tau* = 0.801, *P* = 0.0026), lift (Kendall’s *Tau* = 0.738, *P* = 0.0056), and the lift-on-drag ratio (Kendall’s *Tau* = 0.609, *P* = 0.0221) on shells 1–3 increased with increasing flow rate, but never did lift forces surpass drag forces.

### Drag and lift with rotation

The rag forces increased with increasing rotation angle of the shells against the flow (Pearson *r* = 0.671, *P* = 0.0062; drag _0°_ = 62.23 ± 25.84, drag _45°_ = 71.38 ± 20.72, drag _90°_ = 117.24 ± 30.50 μN) (Fig. 4a), obviously because the cross-sectional areas of the objects increased with rotation angle (*r* = 0.625, *P* = 0.0137). Unlike drag, lift forces decreased with increasing rotation (Kendall’s *Tau* = − 0.552, *P* = 0.0041; lift _0°_ = 39.07 ± 25.08, lift _45°_ = 25.66 ± 11.13, lift _90°_ = 11.72 ± 15.37 μN) (Fig. 4b). The lift-on-drag ratio decreased significantly with increasing rotation angle of the shell towards the flow (Kendall’s *Tau* = − 0.665, *P* = 0.0005), but was not affected by shell height (Kendall’s *Tau* = 0.210, *P* = 0.4527), the cross-sectional area of the shell (Pearson *r* = − 0.212, *P* = 0.4487), nor by shell shape (Kendall’s *Tau* = − 0.299, *P* = 0.1202).

### Lateral force

As expected from dextral-coiling shells aligned with the flow direction, the shells experienced a slight horizontal shifting force, perpendicular to the flow direction, directed to the left (7.23 ± 2.54 μN) (Fig. 4d). When rotated anticlockwise, the lateral force had a higher intensity and was oriented in the opposite direction (Kendall’s *tau* = − 0.665, *P* = 0.0006), quasi pushing the shells back into the optimal alignment with the flow. There was no significant difference in intensity between 45° (− 24.22 ± 6.26 μN) and 90° (− 28.54 ± 7.35 μN) (*t* = 0.999, *P* = 0.3467) (Fig. 4d). At 0° orientation, shell 1 experienced the highest lateral force (8.6 μN), scaled shells 2 (8.43 μN) and 3 a similar force (7.34 μN) (Fig. 4d). The lateral forces increased with increasing flow velocity (Kendall’s *Tau* = 0.866, *P* = 0.0012).

### Laminar to vortical flow transition

**Fig. 5:**
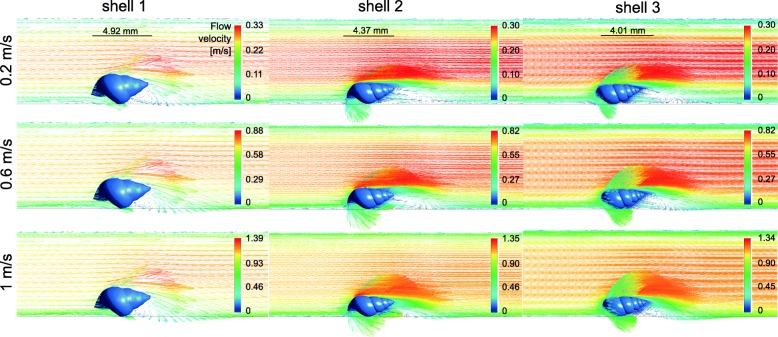
Lateral view of the flow environment around the STL shell models 1–3 at three different flow velocities. Original flow direction in the channel from left to right

**Fig. 6:**
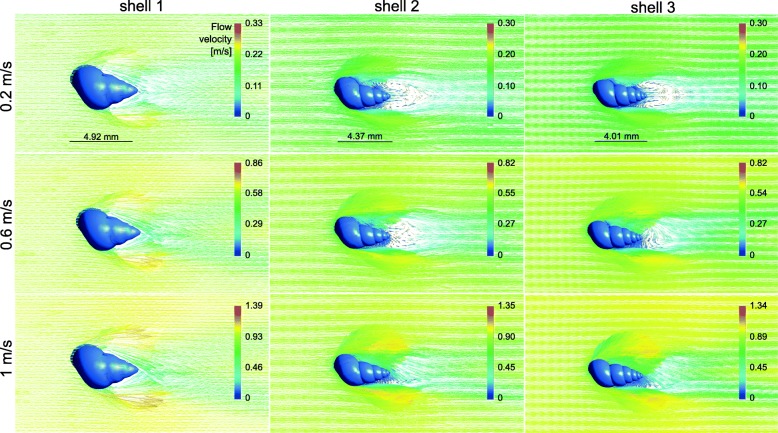
Top view of the flow environment around the STL shell models 1–3 at three different flow velocities. Original flow direction in the channel from left to right

A transition from laminar to vortical flow was observed for all three shell models at all tested flow velocities (Figs. 5 and 6). A flow separation in layers of different flow velocities was present on the upper side of the shells; the closer to the shell, the lower was the flow rate of a layer. The starting point of the flow separation was located far more anterior on the upper shell side and the flow deceleration was strongest at the ventral side of the shells, where the flow perturbations were located (Fig. 5). The widths of the zones including vortices were proportional to the widths of the shells (Fig. 6).

### Flow tank experiment

**Fig. 7:**
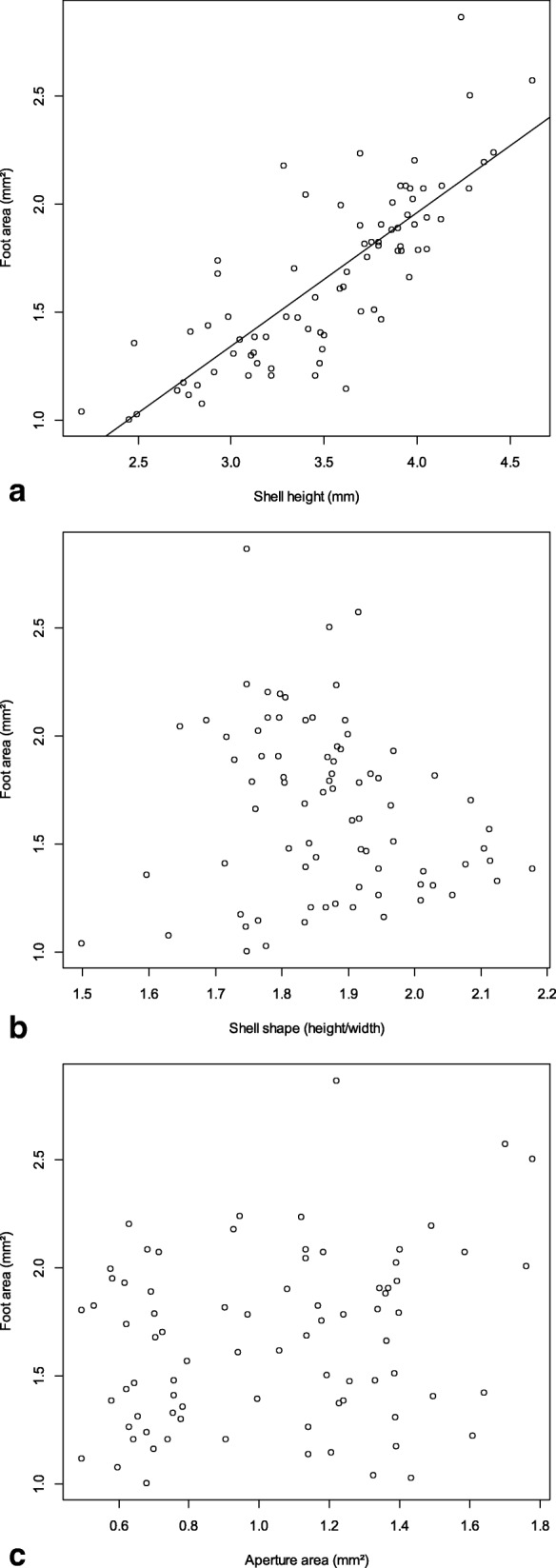
Foot area of the *P. antipodarum* individuals used in the flow tank experiment plotted against shell height (**a**), shell shape (**b**) and aperture area (**c**)

Shell height varied between 2.19 and 4.41 mm (3.47 ± 0.52 mm – mean ± sd) across all specimens, shell width between 1.40 and 2.53 mm (1.90 ± 0.30 mm). Shell height and shell shape did not differ significantly between lake and spring brook sites (*t*_height_ = 0.8902, *P* = 0.3730; *t*_shape_ = 1.7338, *P* = 0.0870). The aperture area was positively correlated with shell height (Kendall’s *Tau* = 0.192, *P* = 0.012) and shell width (*Tau* = 0.184, *P* = 0.016), but not with shell shape (*Tau* = − 0.011, *P* = 0.885). The maximum foot size was only correlated with shell height (Fig. 7a), but not with shell shape (Fig. 7b), the interaction of shell height and shape, aperture area (Fig. 7c), habitat type, population origin or the interaction of aperture surface and population origin (Table 3). Thus, our hypothesis that foot size could be predicted by shell shape or aperture size was rejected. Both, the aperture and foot areas corrected for shell height did not vary between our lake and spring sites (*t*_relative aperture_ = 0.0003, *P* = 0.9998; *t*_relative foot_ = − 1.1115, *P* = 0.2698). The mean detachment speed was 0.739 ± 0.135 m/s and ranged from 0.46 to 0.96 m/s. Finally, the LM with detachment speed as response variable did not retain any of the explanatory variables, i.e. detachment was not determined by shell height or shape, foot size, aperture area, habitat or population origin.

**Table 3:** Parameter estimates of the optimal linear model for the foot area in the flow tank experiment. SE = standard error

	Estimate	SE	*t*	*P*
Intercept	−1.310	0.154	8.490	< 0.0001
Shell height	2.46 × 10^−4^	1.87 × 10^−5^	− 13.173	< 0.0001
Shell shape	−0.106	0.076	1.383	0.1710

Residual standard error = 0.0859, df = 76, multiple R^2^ = 0.6958, adjusted R^2^ = 0.6878

## Discussion

Freshwater gastropods commonly show larger and more globular shell shapes with a larger aperture area in lotic vs lentic habitats. Here, we used the generalist snail *P. antipodarum* to investigate the potential hydrodynamic advantages of these counterintuitive high-flow shell morphologies using CFD simulations and a flow tank experiment. We hypothesized that these shell morphologies reduce the dislodgement risk by allowing a larger foot for increased attachment strength and by reducing the ratio of lift vs drag forces acting on the shells.

### Higher drag and lift forces on larger and wider shells

As expected, the drag forces measured in our CFD simulations increased with the size of the shells, and were highest for the most globular shell, although of similar magnitude for the intermediate and slender shell morphs when scaled to the same height. The drag was also always the highest force experienced by the shells compared to the lift or lateral forces. Our hypothesis that the presence of wider shells with shorter spires in lotic environments could be explained by an adaptation against lift rather than drag forces was rejected. The lift-on-drag ratio was even the highest for the most globular shell. Globular shells also produced a wide vortical zone underneath the shell.

The starting point of the flow separation was located far more anterior on the upper shell side, unlike starting from the tip as detected by Statzner & Holm [4] by Laser Doppler Anemometry. The flow deceleration was strongest at the ventral side of the shells, where the flow perturbations were located, which matched with the dead water zones, i.e. a zone where the flow velocity fluctuates around 0 m/s, found by Statzner & Holm [4]. It is important to note that in the CFD simulations, the forces on the shells alone were tested without the presence of the snail body carrying the shell. This certainly affected the flow conditions at the boundary layer at the bottom and the lift forces acting on the shells as the presence of a head and foot under the shell would prevent water passing underneath it, thus increasing upwards directed lift forces resulting from the acceleration of the flow along the upper shell surface alone. Similarly, the vortices that were observed under the ventral side of the shells would probably be reduced. In future experiments, it would therefore be interesting to simulate the flow around shells including the snail bodies; however, it will not be straightforward to account for the soft and adaptive body consistency.

### Drag and lateral forces explain rheotaxis

*P. antipodarum* is known to show rheotactic behavior, i.e. to align its body with the direction of a current ([52], personal observations). As drag and lateral forces increase with increasing rotation of the snail out of the flow, rheotaxis could be a way to decrease the dislodgement risk through those forces. In contrast, lift decreased with increasing rotation, possibly mitigating the effects of drag and lateral forces.

### Foot size and dislodgement speed uncorrelated to shell shape

In the flow tank experiment, the foot size was only predicted by the size of the shell, not shell shape or aperture size. Although empirical evidence is scarce, researchers have long used the aperture area of gastropods as a proxy for the foot area (e.g. [14]). Unlike the soft body it is indeed easier and more convenient to measure the hard, external shells in gastropods, which can be done on fixed specimens or empty shells. Our findings show that this assumed aperture/foot area correlation should be used with caution and cannot be generalized for all aquatic gastropod species.

The dislodgement speed varied between 0.46 and 0.96 m/s, which is within the flow velocity range experienced by this snail in the field [22, 23]. The minimum dislodgement speed was twice as high as the highest flow rate 4.5–5 mm long *P. antipodarum* could stay attached in preliminary trials by Levri & Clark [50]. The range of dislodgement speeds was similar to the 0.33–0.86 m/s range measured in flow tank experiments for various other freshwater gastropods except limpet-shaped species, which could resist currents that were almost three times as high (e.g. [16–18, 53]).

The dislodgement speed could not be explained by shell, aperture and foot size, nor by shell shape or the habitat type and population the snails were originally collected from. If not by shell morphology or foot size, aquatic gastropods likely present other adaptive traits to reduce the dislodgement risk by flow, for instance through behaviour or increasing the stickiness of the pedal mucus. For example, variation in rheotaxis and dispersal was demonstrated between same sized *P. antipodarum* from different invasive clones [52]. Pedal mucus plays an important role for the adhesion of gastropods due to its viscoelasticity (reviewed by [54, 55]). Some aquatic gastropods are known to change the adhesion tenacity of their pedal mucus through changing its composition when alternating between active mobile and glued static states (e.g. in *Littoraria irrorata* [56] or *Lottia limatula* [57]). During fieldwork in New Zealand we encountered two river populations of *P. antipodarum*, which apparently had a stickier mucus and crawled on the exposed faces of stones in contrast to their conspecifics in other localities. Thus, there exists a potential for aquatic gastropods to counter the variations in flow and decreasing dislodgement risks by varying these traits.

## Conclusions

The integration of our multi-disciplinary approaches provided further insights into the adaptations of shells to flow. Otherwise, our conclusions would have remained ambiguous or even different. Our hypothesis that the controversial presence of globular shelled gastropods in lotic environments could be explained by an adaptation against lift rather than drag forces was rejected. Both, drag and lift forces were stronger on globular compared to slender shells. This explains why slender shells showed higher fitness, measured as the number of brooded embryos, compared to globular ones in high flow environments in both native and invasive populations [22, 23]. An absolutely or relatively larger foot size did not explain the presence of more globular shells in lotic environments, either. Foot size in *P. antipodarum* was only predicted by the size of the shell, not by shell shape or aperture size, showing that the assumed aperture/foot area correlation should be used with caution and cannot be generalized for all aquatic gastropod species. Finally, shell morphology and foot size were not related to dislodgement speed in our flow tank experiment. Hence, other traits must play a major role in decreasing the dislodgement risk in stream gastropods, e.g. specific behaviours or pedal mucus stickiness. Although we did not find that globular shells are adaptations for reducing the dislodgement risk, we cannot rule out that they are still flow related adaptations. For instance, globular shells are more crush-resistant and therefore perhaps adaptive in terms of diminishing damage caused by tumbling after dislodgement as suggested by [11]. Globular shells of gastropods are also known to be an adaptation against crush-type predators (e.g. [58, 59]). Therefore, the increase in globularity of the shells in lotic habitats represents maybe an adaptation against habitat specific predators as recently demonstrated for *Lithasia geniculata* [12]. We conclude that in aquatic gastropods the relationships of shell morphology and habitat are more complex than assumed. Flow velocity certainly does not exert the only selective pressure as already suggested by our foregoing field studies [22, 23].

## Additional file

Additional file 1: **Table S1.** Reynolds numbers (Re) for the flow in the channel and for the shell models at 0° rotation from the flow direction in the computational fluid dynamic simulations. (DOCX 12 kb)
