# Supplementary material for: Testing the adaptive value of gastropod shell morphology to flow: a multidisciplinary approach based on morphometrics, computational fluid dynamics and a flow tank experiment
Source: Zoological Lett. 2019 Jan 18;5:5. doi: 10.1186/s40851-018-0119-6 (PMC6337808; doi:10.1186/s40851-018-0119-6)
Supplement: Supplementary file 1 — Table S1. Reynolds numbers (Re) for the flow in the channel and for the shell models at 0° rotation from the flow direction in the computational fluid dynamic simulations. (DOCX 12 kb) [file 40851_2018_119_MOESM1_ESM.docx]

**Additional file 1: Table S1.** Reynolds numbers (Re) for the flow in the channel and for the shell models at 0° rotation from the flow direction in the computational fluid dynamic simulations

|  | Flow velocity (m/s) | Re |
| --- | --- | --- |
| Channel | 0.2 | 2793 |
| Channel | 0.6 | 8381 |
| Channel | 1 | 13968 |
| Shell 1 | 0.2 | 620 |
| Shell 1 | 0.6 | 488 |
| Shell 1 | 1 | 396 |
| Shell 2 | 0.2 | 1860 |
| Shell 2 | 0.6 | 1464 |
| Shell 2 | 1 | 1188 |
| Shell 3 | 0.2 | 3100 |
| Shell 3 | 0.6 | 2440 |
| Shell 3 | 1 | 1980 |
